# INEQUALITIES IN OLDER ADULTS’ LIFELONG LEARNING: HOW SOCIO-ECONOMIC STATUS SHAPES PARTICIPATION AND BENEFITS

**DOI:** 10.1093/geroni/igae098.0093

**Published:** 2024-12-31

**Authors:** Shu Yee Chin, Jolin Chua, Nathan Widjaja, Shannon Ang, Rahul Malhotra

**Affiliations:** Nanyang Technological University, Singapore, Singapore; Nanyang Technological University, Singapore, Singapore; Nanyang Technological University, Singapore, Singapore; Nanyang Technological University, Singapore, Singapore; Duke-NUS Medical School, Singapore, Singapore

## Abstract

Although lifelong learning has been increasingly utilized to promote active ageing, research increasingly suggests that older adults with lower socio-economic statuses (SES) may face unique barriers to participation in these programs. Simultaneously, we know little about whether SES moderates the well-being benefits of lifelong learning programs. Using two waves of data (2016-17; 2019) from a nationally representative sample of Singaporeans aged 60 and above (n = 2,508), this study investigates whether older adults’ SES shapes their participation and benefits reaped from lifelong learning programs. Firstly, we used a multinomial logistic regression model to examine the relationships between SES and participation in job-related and nonjob-related lifelong learning programs. Secondly, we used lagged dependent variable regression analyses with inverse probability weight adjustments to examine whether SES moderated the relationship between lifelong learning participation and quality of life, while addressing potential selection biases into lifelong learning participation. Findings indicate that older adults with higher educational attainment and occupational prestige had greater participation in both job- and nonjob-related lifelong learning. Moderation analyses further showed that the effects of nonjob-related learning on quality of life were only significant for those with lower SES. In summary, these findings suggest that although lower-SES older adults benefit more from participation in lifelong learning programs, they are also less likely to participate in such programs. Results highlight the need to make programs more accessible to lower-SES older adults. Future research should continue to investigate the mechanisms through which SES moderates the benefits of lifelong learning for well-being outcomes.

